# A 17-cm ileocecal inflammatory myofibroblastic tumor with severe inflammatory syndrome in a child: a case report

**DOI:** 10.1093/jscr/rjag996

**Published:** 2026-11-02

**Authors:** Junxiao Wang, Jianping Zhang, Guihua Le, Chuanqi Ren, Yihan Zhang, Ezhang Liu

**Affiliations:** The First Ward of the Department of General Surgery, The Second Hospital of Sanming, No. 513, Rongkang East Road, Yong’an City, Sanming, Fujian 366000, China; Department of Imaging, The Second Hospital of Sanming, No. 513, Rongkang East Road, Yong’an City, Sanming, Fujian 366000, China; Department of Pathology, The Second Hospital of Sanming, No. 513, Rongkang East Road, Yong’an City, Sanming, Fujian 366000, China; The First Ward of the Department of General Surgery, The Second Hospital of Sanming, No. 513, Rongkang East Road, Yong’an City, Sanming, Fujian 366000, China; The First Ward of the Department of General Surgery, The Second Hospital of Sanming, No. 513, Rongkang East Road, Yong’an City, Sanming, Fujian 366000, China; The First Ward of the Department of General Surgery, The Second Hospital of Sanming, No. 513, Rongkang East Road, Yong’an City, Sanming, Fujian 366000, China

**Keywords:** inflammatory myofibroblastic tumor, ileocecal, pediatric, giant tumor, paraneoplastic inflammatory syndrome, case report

## Abstract

We present a case of a giant ileocecal inflammatory myofibroblastic tumor (IMT), which represents one of the largest pediatric colorectal IMTs reported in the literature. An 11-year-old girl presented with a 1-day history of abdominal pain and developed fever (up to 102.2°F). Laboratory evaluation revealed severe anemia (hemoglobin, 53 g/L) and marked systemic inflammatory abnormalities consistent with paraneoplastic inflammatory syndrome. Exploratory laparotomy revealed a 17 × 17 × 8 cm mass intimately adherent to the ileocecal region. Histopathological examination confirmed IMT with focal ALK positivity. The inflammatory and hematologic abnormalities improved rapidly following complete tumor resection.

## Introduction

Inflammatory myofibroblastic tumor (IMT) is a rare mesenchymal neoplasm of intermediate biological potential, characterized by myofibroblastic spindle cells and a mixed inflammatory infiltrate [1]. IMT accounts for ~ 0.04%–0.7% of pediatric tumors, with the lung being the most common primary site, followed by the abdomen and retroperitoneum [2]. The World Health Organization classifies IMT as an intermediate neoplasm that rarely metastasizes [3].

Pediatric colorectal IMT is exceptionally rare. A recent systematic review identified only 53 pediatric colorectal IMTs reported worldwide through January 2025. Among cases with a specified location, the rectum and ascending colon were the most frequently involved sites, whereas only four cecal tumors were reported [4]. Ileocecal IMT may present with abdominal pain, fever, anemia, or a palpable abdominal mass and may clinically and radiologically mimic Burkitt lymphoma and other pediatric abdominal tumors, posing a substantial diagnostic challenge [4, 5].

We herein report a giant 17-cm ileocecal IMT in an 11-year-old girl who presented with severe systemic inflammatory manifestations consistent with paraneoplastic inflammatory syndrome (PIS), including high fever, severe anemia, marked thrombocytosis, elevated C-reactive protein (CRP), and hypoalbuminemia. Histopathological examination confirmed IMT, and complete resection with negative margins was achieved. The unusual combination of an exceptionally large tumor, a rare ileocecal location, and prominent systemic inflammatory manifestations makes this case clinically noteworthy. It also illustrates that ileocecal IMT should be considered in the differential diagnosis of large pediatric abdominal masses and that tumor-associated inflammatory abnormalities may improve rapidly after complete resection.

## Case report

### Patient information

An 11-year-old girl was admitted on 17 June 2026, with a 1-day history of persistent, dull, generalized abdominal pain, without vomiting, diarrhea, or hematochezia (Table 1).

**Table 1:** Hospital course timeline.

**Time point**	**Event**
1 day before admission	Onset of abdominal pain
Hospital day 1 (admission)	Hemoglobin: 53 g/L; platelets: 1063 × 10^9^/L; C-reactive protein: 197.89 mg/L; albumin: 22.6 g/L
Hospital day 2	Transfusion of 2 units of leukocyte-reduced packed red blood cells; contrast-enhanced CT of the whole abdomen; complete gastrointestinal barium fluoroscopy; ascitic fluid aspiration with cytology for malignant cells
Hospital day 4	Transfusion of 2 units of leukocyte-reduced packed red blood cells, intravenous infusion of human albumin injection
Hospital day 6	Onset of fever (T 38°C/100.4°F)
Hospital day 7	Fever (T 39°C/102.2°F), blood culture
Hospital day 10	Multidisciplinary consultation (MDT): the primary consideration is IMT; Burkitt lymphoma is not ruled out.
Postoperative day 0	Exploratory laparotomy with ileocecal resection
Postoperative day 1	Pathological diagnosis: IMT
Postoperative day 8	Body temperature: 36.7°C/98.06°F; hemoglobin: 76 g/L; white blood cells: 7.03 × 10^9^/L; platelets: 715 × 10^9^/L; albumin: 30.4 g/L; C-reactive protein <10 mg/L
Postoperative day 13	Discharge
Postoperative day 20	Hemoglobin: 104 g/L; white blood cells: 5.92 × 10^9^/L; platelets: 382 × 10^9^/L

### Clinical findings

On admission, her body temperature was 39°C. Physical examination revealed pallor of the lips and palpebral conjunctivae. Abdominal examination revealed a palpable mass in the right lower abdomen, measuring ~ 15 × 10 cm. It was firm and mobile, with mild tenderness and poorly defined margins.

### Laboratory findings

Laboratory evaluation revealed severe anemia (hemoglobin, 53 g/L), leukocytosis (white blood cell count, 14.99 × 10^9^/L), marked thrombocytosis (platelet count, 1063 × 10^9^/L), a markedly elevated CRP level (197.89 mg/L), hypoalbuminemia (albumin, 22.6 g/L), and a lactate dehydrogenase (LDH) level of 94 U/L. Serum alpha-fetoprotein (AFP), carcinoembryonic antigen, and β-human chorionic gonadotropin (hCG) levels were within the normal ranges. Cytological examination of the ascitic fluid revealed no malignant cells, and blood cultures were negative.

### Imaging studies

Contrast-enhanced computed tomography (CT): the mass was located in the right lower abdomen at the ileocecal region. Dynamic contrast-enhanced scanning demonstrated significant heterogeneous progressive enhancement. The demarcation between the lesion and adjacent bowel loops was poorly defined. Prominent arteriovenous vessels were visualized around the lesion (Fig. 1).

**Figure 1:**
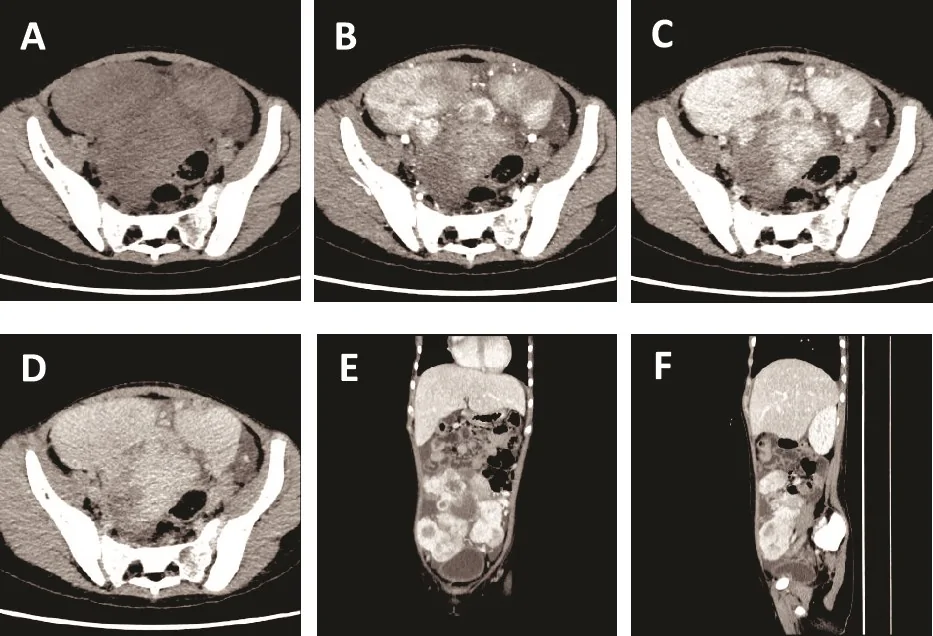
Multiphasic contrast-enhanced CT findings of inflammatory myofibroblastic tumor. (A) Non-contrast phase; (B) arterial phase; (C) portal venous phase; (D) equilibrium phase; (E) coronal portal venous phase; and (F) sagittal portal venous phase.

### Diagnostic challenges and differential diagnosis

The differential diagnosis prioritized ovarian malignant germ cell tumor and abdominal Burkitt lymphoma, while also considering gastrointestinal stromal tumor and IMT.

### Preoperative management

A total of 4 units of leukocyte-depleted packed red blood cells were transfused preoperatively. Surgery was planned after hemoglobin recovery.

### Surgical procedure

A giant mass was identified, covered by omentum with prominent hypertrophied vessels on its surface. The mass was intimately adherent to the ileocecal region with an intact capsule. Ileocecal resection was performed followed by ileocolic anastomosis (Fig. 2).

**Figure 2:**
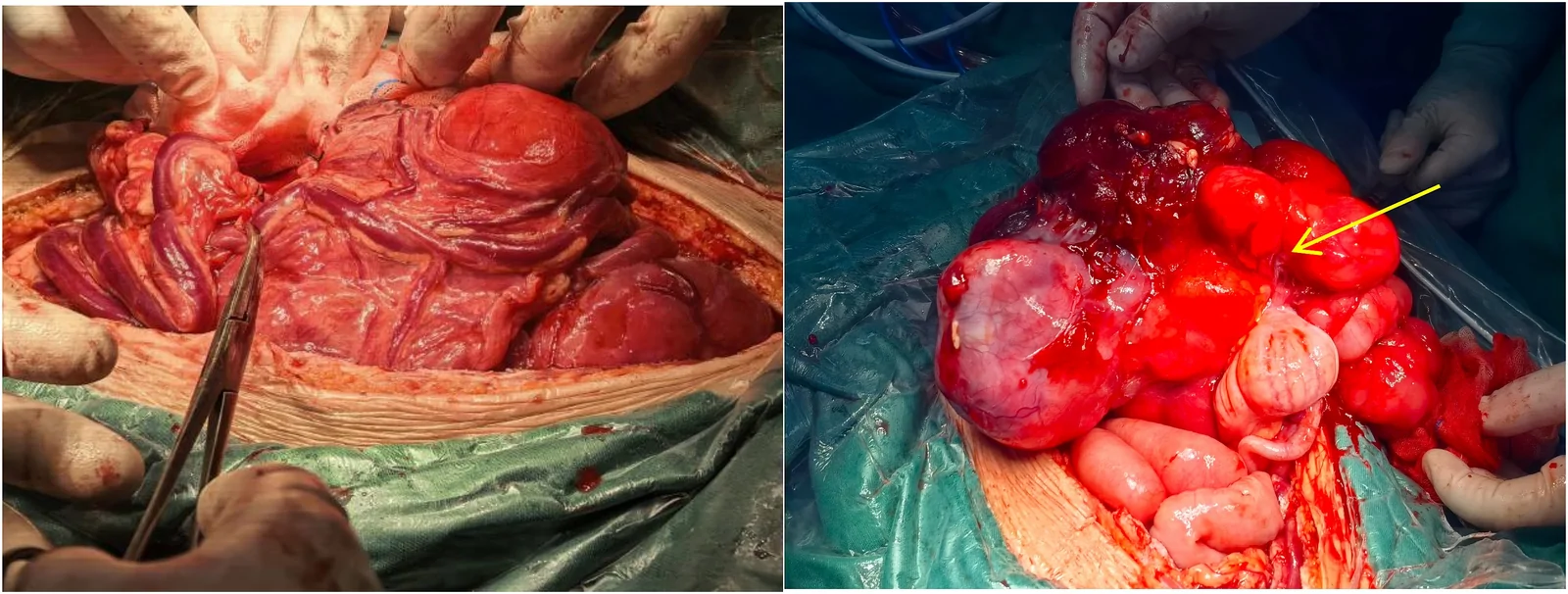
Intraoperative findings of intestinal inflammatory myofibroblastic tumor. (A) Hypertrophic and enlarged feeding vessels supplying the inflammatory myofibroblastic tumor; (B) inflammatory myofibroblastic tumor adherent to the ileocecal region (arrow indicates the site of adhesion).

### Pathological reports

Ileocecal mass: a lobulated, nodular mass was identified on the serosal surface of the intestinal wall, measuring 17 × 17 × 8 cm. The tumor was composed of spindle-shaped fibroblasts and myofibroblasts arranged in fascicular and interlacing patterns, with abundant lymphocytic and plasma cell infiltration in the stroma. The tumor cells showed no significant atypia. Mitotic figures were rare (3 per 4 mm^2^). Pericolic lymph nodes (0/11) showed no evidence of tumor metastasis. Tumor cells were positive for vimentin, SMA, and desmin, with focal positivity for ALK using both the D5F3 and 5A4 antibody clones. Partial expression of AE1/AE3 and P16 was noted. CD117, DOG-1, S100, STAT6, and CD30 were negative, supporting IMT and excluding major histological mimics. The Ki-67 index was 12% in hotspots (Fig. 3).

**Figure 3:**
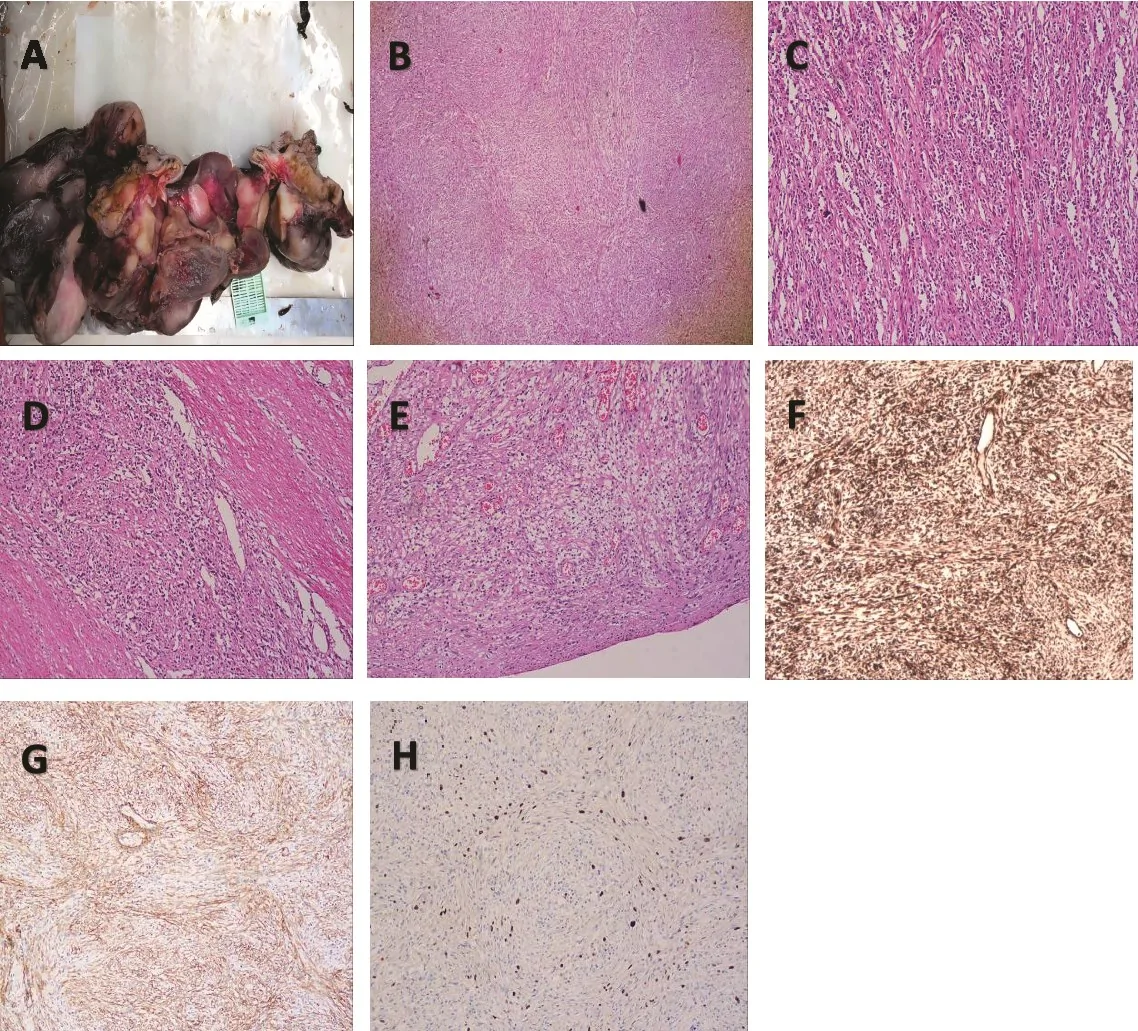
Clinicopathological features and immunohistochemical analysis of inflammatory myofibroblastic tumor. (A) Gross specimen showing a lobulated, well-circumscribed nodular mass on the serosal surface of the intestinal wall with a solid cut surface; (B) spindle-shaped tumor cells arranged in fascicular and interlacing patterns (H&E); (C) prominent lymphoplasmacytic infiltration within the tumor stroma (H&E); (D) tumor infiltrating the muscularis propria of the intestinal wall (H&E); (E) well-defined tumor border at the serosal surface (H&E); (F) immunohistochemistry showing cytoplasmic and membranous positivity for ALK (D5F3 clone); (G) immunohistochemistry showing positive expression of Desmin in tumor cells; and (H) Ki-67 proliferation index of 12% in hotspot areas.

### Follow-up and outcomes

By postoperative day 8, the patient’s body temperature, white blood cell count, and CRP level had normalized (98.1°F, 7.03 × 10^9^/L, and < 10 mg/L, respectively). Her hemoglobin level had increased to 76 g/L, albumin level to 30.4 g/L, and platelet count had decreased to 715 × 10^9^/L. By postoperative day 20, her hemoglobin level had further increased to 104 g/L, while her platelet count had decreased to 382 × 10^9^/L, indicating continued improvement in the hematologic abnormalities without additional treatment. Her body temperature remained normal after surgery. Given the complete surgical resection with negative margins (R0) and the absence of lymph node metastasis (0/11), no adjuvant therapy was administered. At the most recent follow-up, 2 months after surgery, the patient remained clinically well, without recurrent fever, abdominal pain, or other tumor-related symptoms.

## Discussion

The most notable features of this case are the exceptionally large tumor size (17 cm), its ileocecal location, and the presence of severe PIS. In the classic series of 84 extrapulmonary IMT cases reported by Coffin, the mean diameter was 6.4 cm, with abdominal tumors tending to be the largest, though most commonly arising from the mesentery and omentum (36/84) [6]. Of particular note, the patient exhibited pronounced systemic inflammatory abnormalities, including fever, severe anemia, leukocytosis, marked thrombocytosis, markedly elevated CRP, and hypoalbuminemia, a constellation of findings characteristic of severe PIS in patients with solid tumors [7].

In the present case, the attribution of the systemic inflammatory abnormalities to PIS was supported by their close temporal relationship with the tumor and their rapid improvement following tumor resection. Before surgery, the patient presented with a fever of up to 39°C (102.2°F), severe anemia (hemoglobin, 53 g/L), leukocytosis, marked thrombocytosis (1063 × 10^9^/L), a markedly elevated CRP level (197.89 mg/L), and hypoalbuminemia (22.6 g/L), collectively indicating a pronounced systemic inflammatory response. Following complete tumor resection, her body temperature, white blood cell count, and CRP level normalized by postoperative day 8, while the albumin level increased and the platelet count progressively decreased without additional treatment. These findings support a tumor-associated inflammatory process. Although infection, tumor-related blood loss, and impaired nutritional status could also have contributed to individual abnormalities, their concurrent improvement after tumor removal favored PIS as the unifying explanation. Mechanistically, interleukin (IL)-6 has been proposed as a major mediator of PIS by promoting hepcidin production, stimulating thrombopoietin-mediated megakaryocyte proliferation, increasing positive acute-phase proteins such as CRP, and reducing negative acute-phase proteins such as albumin [8–10]. However, serum IL-6 was not measured in this patient; therefore, its involvement remains mechanistically plausible but unconfirmed. It is worth noting that tocilizumab has shown potential as a symptomatic treatment for severe PIS. In a retrospective study of 35 patients with solid tumors, it was associated with resolution of paraneoplastic fever and improvement in several inflammatory abnormalities; performance status improved in 31% of patients [7]. In selected patients whose inflammatory condition initially precluded surgery, temporary control of PIS with tocilizumab facilitated subsequent surgical treatment.

In this case, LDH was not elevated, no tumor cells were identified in the ascitic fluid, and CT demonstrated heterogeneous progressive enhancement with prominent feeding vessels, which also differs from the homogeneous mild-to-moderate enhancement pattern typical of lymphoma. These findings therefore do not support this diagnosis [11]. Meanwhile, normal AFP and β-hCG levels made several secreting germ cell tumor subtypes less likely [12].

Complete surgical resection (R0) is the gold standard for the treatment of IMT. The recurrence rate in patients with positive margins was significantly higher than in those with negative margins (40% vs. 0%, *P* = .04) [4]. Although pediatric IMT has an overall recurrence rate near 20%, colorectal tumors appear to recur less often, at ~ 11% [4].

### Molecular subtyping and prospects for targeted therapy

Approximately 50%–60% of IMTs harbor ALK gene rearrangements, which represent the most common molecular alterations in IMT [13]. The EORTC 90101 CREATE study indicated that crizotinib achieved an objective response rate of 50% in ALK-positive IMT patients [14]. ROS1 and NTRK3 are among the targetable driver genes detectable in ALK-negative IMT [3]. A recent institutional molecular profiling study identified a rare VCAN–IL23R fusion in one IMT case [15].

In this case, ALK status was assessed solely by immunohistochemistry, without further confirmation of ALK gene rearrangement or identification of the specific fusion partner by FISH or RNA sequencing [16].

## Conclusion

Ileocecal IMT can be considered in children with a large hypervascular abdominal mass and systemic inflammatory abnormalities. Complete resection with negative margins remains the preferred treatment for localized disease. Long-term surveillance is required, and RNA-based fusion testing may guide targeted therapy in recurrent, unresectable, or metastatic disease.

## Supplementary Material

Supplementary_material_rjag996

## Data Availability

All data generated or analyzed during this study are included in this published article.

## Patient perspective

As the parents of the patient, we were very worried about our daughter’s abdominal mass after she was admitted to the hospital. We were afraid that it might be diagnosed as a malignant tumor. When she developed a fever, we felt even more overwhelmed and anxious. After the medical team’s thorough evaluation and treatment, we agreed to proceed with surgery. The surgery was very successful, and our daughter recovered well without any complications. We would like to express our heartfelt gratitude to the medical team. We also consent to sharing our daughter’s medical records and treatment course with other physicians.
